# Ultrasound-based classification and rehabilitation of biceps femoris T-junction injuries

**DOI:** 10.3389/fspor.2026.1735177

**Published:** 2026-02-03

**Authors:** Kevin Cronin, Fearghal Kerin

**Affiliations:** 1 School of Medicine, University College Dublin, National University of Ireland, Dublin, Ireland; 2 Kerin Performance, Dublin, Ireland

**Keywords:** classification, hamstring, injury, T-junction, ultrasound

## Abstract

The T-junction represents the interface between the long and short heads of the biceps femoris, comprising superficial myo-tendinous and deeper myo-aponeurotic connective-tissue components. Injury to this region is frequently under-recognised on MRI and associated with prolonged recovery and recurrence. A new ultrasound-based classification system is proposed, defining five subtypes according to structural involvement, myofascial extension, haematoma formation, and dynamic behaviour during resisted contraction. This classification system does require testing *in vivo* to establish validity. Ultrasound provides superior spatial resolution and allows direct assessment of tendon continuity and motion between the long and short heads. Rehabilitation strategies are aligned with injury subtype and guided by ultrasound findings rather than time alone. The approach integrates early protection and reactivation with progressive restoration of intermuscular coordination, strength, and high-speed load tolerance. The framework provides a structured method for diagnosis, prognosis, and rehabilitation planning in athletes with T-junction injuries of the biceps femoris.

## 1 Introduction

Hamstring injuries remain one of sport's most persistent clinical challenges. Recent research has shifted attention toward subtypes defined by specific structural involvement, including intramuscular tendon injuries, free-tendon injuries, and the increasingly recognised biceps femoris T-junction tears. First described by Entwisle in 2017 (1), the T-junction denotes the distal musculotendinous junction where the long and short heads of the biceps femoris converge, forming a complex, dual-innervated region exposed to high mechanical stress and strain. These lesions may appear less extensive on MRI than intraoperative findings subsequently reveal, as demonstrated by Kayani and colleagues (2). Entwisle reported reinjury rates of up to 57%, and subsequent studies suggest that these injuries are not only more prone to recurrence but may also exacerbate during rehabilitation, reflecting the challenge of matching progression to tissue capacity (1, 3).

Current MRI-based systems, such as the British Athletics Muscle Injury Classification (BAMIC), provide valuable structure for communication and represent an exceptional tool for guiding rehabilitation and improving outcomes (4, 5). However, these frameworks were not designed to accommodate the complex and distinct anatomy of the T-junction, which spans tendon limbs and connective-tissue planes. As different connective-tissue types respond and adapt to loading, and remodel, at different cadences, a more bespoke system could in theory assist clinicians in designing and periodizing rehabilitation with greater precision. The London International Consensus recognised that existing frameworks remain relatively broad and are not specific to individual hamstring muscles (6), leaving T-junction injuries outside their scope. The authors of the BAMIC could argue that this is reasonable, given that they, like Kerin et al. (7)., found no clear difference between T-junction and non–T-junction cases (8). Nevertheless, the distinction remains clinically relevant, as (5) demonstrated that aligning rehabilitation strategies with the precise structure injured can meaningfully alter outcomes.

Recent experimental work has shed new light on the unique behaviour of the components of the T-junction. Nakao et al. (9) showed that the junction serves as a critical site of transverse force transmission between the long and short heads of the biceps femoris, with the short head playing a key role in regulating long head strain. Disruption of this interface alters load distribution and intermuscular coordination (10)— a dynamic phenomenon not reliably captured on static MRI (11). In contrast, ultrasound allows direct assessment of tendon continuity and asynchronous motion (dynamic structural integrity), offering a more functionally relevant view of healing and adaptation (12, 13.

As highlighted by Balius et al. (14), muscle injuries vary significantly in structure, behaviour, and healing characteristics between muscle groups, limiting the clinical utility of a single, unified classification. They argued that certain muscles—particularly those with complex connective-tissue interfaces—may warrant muscle-specific subclassifications to improve diagnostic precision and prognostic accuracy.

This supports the need for a more granular approach. If T-junction injuries represent a distinct structural and functional entity, they warrant their own classification system. Thus, this paper presents a novel ultrasound-based framework for T-junction injuries, organised from Type 1 to Type 5 in increasing structural severity, to improve diagnostic precision, guide prognosis, and inform progressive rehabilitation planning based on the extent and depth of tissue involvement.

## 2 Terminological and structural framework of the T-junction

Building on earlier anatomical descriptions of the distal T-junction (1, 15), Cronin et al. (13) demonstrated, for the first time, the use of dynamic ultrasound to visualise its two distinct components *in vivo*. The superficial myotendinous connective-tissue component represents the interface where muscle fibres insert directly into tendon or aponeurotic tissue. These two components are closely integrated but can be differentiated structurally and functionally.

The deeper myo-aponeurotic connective-tissue component, often termed the “zipper” region (1, 15), corresponds to the confluence of the epimysial surfaces of the long and short heads of the biceps femoris. It is not a classical musculotendinous junction or an intratendinous extension, as might be assumed if applying the BAMIC framework beyond its intended scope. Rather, it represents the meeting point of two opposing epimysial surfaces—aponeurotic layers in functional terms—forming a composite connective-tissue interface between the two heads (1, 16). Instead of a discrete tendon insertion, this region forms a continuous myo–myo–aponeurotic interface linking both heads through the extracellular matrix. Functionally, it acts as a key site of transverse load transfer within the distal biceps femoris and likely underpins the complex injury behaviour and high recurrence rate observed in T-junction lesions.

A composite intermuscular interface, the T-junction represents a region where the long and short heads interdigitate through shared connective tissue—a hybrid of aponeurotic and musculotendinous structures.

## 3 Role of ultrasound

Understanding the structural complexity of the T-junction is critical when interpreting imaging findings. Poor outcomes and high recurrence rates associated with these injuries, coupled with the increasing accessibility of high-resolution ultrasound, have intensified interest in its use as a complementary imaging modality. While MRI remains valuable for comprehensive assessment, ultrasound provides superior spatial resolution for superficial connective tissue and, importantly, enables real-time evaluation of the junction's dynamic behaviour. As described in a recent review, dynamic ultrasound can identify tendon gapping, loss of continuity, and asynchronous motion between the long and short heads during resisted contraction—features that provide functional insight into tissue integrity and coordination, yet remain invisible to static MRI (12).

## 4 Spatial resolution

Modern musculoskeletal ultrasound systems typically operate above 10 MHz, offering spatial resolutions close to 200 micrometres—substantially superior to MRI for superficial soft tissues. This precision has practical clinical value: Alkhalifah (17) demonstrated that ultrasound outperforms MRI in sensitivity and specificity for small peripheral nerve lesions, underscoring its advantage in resolving fine structural detail. Similar observations have been reported in musculoskeletal imaging, where high-frequency transducers allow accurate delineation of superficial myotendinous and aponeurotic structures with clear correlation to clinical outcomes (12). This is particularly relevant to T-junction injuries, where the myotendinous connective tissue lies just 5–10 mm beneath the skin. At this depth, high-frequency ultrasound provides crisp visualisation of fibrillar structure, vascular response, and early bridging—features that reflect both tissue integrity and biological repair, and which cannot be captured by static MRI.

In addition, recent ultrasound technological advances have improved depth penetration without compromising image quality 18). These developments extend the diagnostic reach of ultrasound to deeper myo-aponeurotic structures, allowing clear visualisation of the “zipper” component of the T-junction (15), typically located 40–50 mm beneath the surface depending on body composition and biceps femoris muscle mass. Image clarity is further enhanced in lean athletic populations, where reduced subcutaneous attenuation enables sharper delineation of connective-tissue architecture and greater diagnostic precision.

## 5 Prognostic implications of tissue involvement

Tears involving the myo-tendinous connective tissue component of the T-junction are generally associated with a poorer prognosis than those confined to the deeper myo-aponeurotic component (19). The myo-tendinous junction is biomechanically more complex and mechanically vulnerable, requiring longer healing periods than the simpler, collagen-based aponeurotic tissue (20, 21).

Healing within the myo-tendinous region involves regeneration of both muscle fibres and tendon interfaces—processes complicated by high mechanical stress and scar formation in this transitional zone. As a result, recovery timelines often exceed 8–10 weeks (2), particularly in larger tears or those involving tendon extension, and require carefully graded rehabilitation to prevent reinjury.

By comparison, aponeurotic tissue, composed predominantly of type I collagen, undergoes a more straightforward repair process with shorter recovery times, unless complicated by significant muscle involvement or strain (22). These structural and biological differences highlight why accurate ultrasound classification is clinically important, as it enables prognosis and rehabilitation planning to be tailored to the specific tissues involved.

## 6 Dynamic evaluation

In contrast to MRI, ultrasound facilitates real-time evaluation of the dynamic interplay between the long and short heads of the biceps femoris during contraction. The following protocol is undertaken:

- Position the transducer transversely over the distal long head of the biceps femoris muscle, where the long head appears medial and the short head lateral; the vertical echogenic striation demarcating these heads constitutes the myoaponeurotic connective tissue.
- Identify the horizontal echogenic striation in the near field representing the myotendinous connective tissue, then evaluate the full T-junction by sliding the transducer superiorly and inferiorly along its path in transverse view, applying minimal pressure to avoid tissue distortion.
- Observe synchronous motion of the heads or dissociation during contraction by applying light counterpressure to the ipsilateral calf with the non-scanning hand, instructing the patient to contract at 25% of their self-gauged maximal effort while externally and internally rotating the tibia; maintain stable transverse transducer positioning over the T-junction.
- If necessary, comparison can be made with the contralateral limb to distinguish normal variability if there is any uncertainty.

As highlighted in recent sports imaging work (12), dynamic ultrasound allows direct visualisation of tissue motion, shear behaviour, and mechanical continuity—features that static MRI cannot capture. Because these injuries often occur during rotational or complex movements, dynamic assessment during functional tasks may seem appealing. However, for consistency and reproducibility, a submaximal isometric contraction at approximately 15° of knee flexion is recommended, as described by Cronin et al. (13). The presence of dissociative movement—often visualised as gapping or separation between the long and short heads—indicates a loss of mechanical continuity across the T-junction. This finding has been proposed as a potential marker for surgical consideration, particularly where asynchronous motion persists despite conservative management or following re-injury (2, 23). Dynamic ultrasound therefore provides a more functionally relevant assessment than static imaging, enabling direct evaluation of tendon integrity, shear displacement, and intermuscular coordination under load—parameters that reflect the mechanical behaviour of the junction and can inform both classification and management decisions.

Ultrasound imaging exhibits excellent intra-observer reliability (ICC range: 0.80–0.99) for quantitative metrics of the musculoskeletal system owing to standardised probe positioning and operator proficiency; however, echo intensity quantification displays heightened variability attributable to subjective time-gain compensation adjustments (24). Inter-observer reliability is good to excellent (ICC range: 0.70–0.97) within sports medicine applications under rigorous protocols, but remains vulnerable to inter-examiner discrepancies in expertise, especially for qualitative evaluations of hypoechoic foci or dynamic manoeuvres Sobolewski et al. (24). These measurement characteristics emphasise the necessity of comprehensive training and methodological standardisation to optimise reproducibility in musculoskeletal diagnostic and prognostic contexts.

## 7 Proposed ultrasound-based classification of biceps femoris T-junction injuries

Given the functional advantages of ultrasound, a structured, ultrasound-based classification system is offered to characterise biceps femoris T-junction injuries more precisely. This framework integrates both static and dynamic findings to define injury severity and guide rehabilitation.

Clinicians should systematically evaluate the following six parameters on ultrasound:

1. Superficial myo-tendinous connective tissue component – the direct interface between muscle fibres and tendon tissue. This region is highly folded and cellular, optimised for contractile force transmission but mechanically vulnerable and prone to delayed healing when disrupted.
2. Deeper myo-aponeurotic connective tissue component – a sheet-like, collagen-dominant structure forming the “zipper” between the long and short heads of the biceps femoris. It provides broad-based load transfer and typically demonstrates more predictable healing behaviour.
3. Extension of the discontinuity into the myofascial connective tissue of the short head (laterally)
4. Extension of the discontinuity into the myofascial connective tissue of the long head (medially)
5. Presence of an intermuscular haematoma – reflecting higher-grade structural disruption and local inflammation between the two heads.
6. Dynamic behaviour under load – evaluation of synchronous vs. asynchronous movement of the biceps femoris heads during resisted isometric contraction, which provides critical insight into tendon continuity and functional stability.

It is recommended that these features are assessed in the transverse imaging plane by an experienced clinician using a high-resolution ultrasound system (12). Handheld or low-specification ultrasound devices should be avoided, as they lack the spatial, axial, and temporal resolution necessary to visualise both the superficial myo-tendinous and deeper myo-aponeurotic components accurately. Furthermore, inadequate temporal resolution prevents reliable detection of asynchronous motion during dynamic assessment.

This classification framework provides a consistent language for reporting, enables meaningful prognostic stratification, and informs the design of targeted rehabilitation programmes that reflect both structural and functional severity.

## 8 T-junction subtype 1

**Figure 1:**
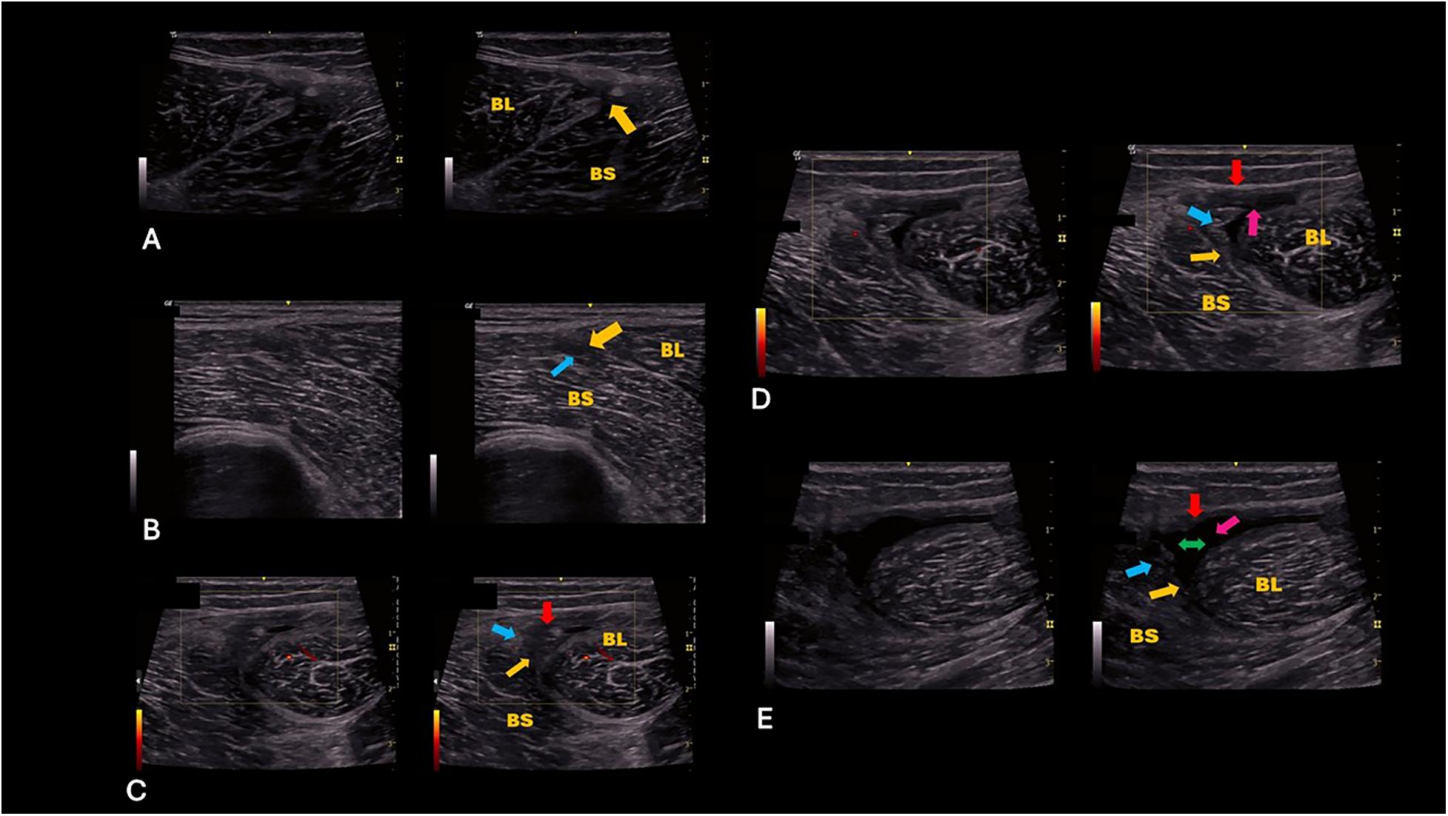
**(A)** A discontinuity to the deep myo-aponeurotic connective tissue component of the T-junction (yellow arrow). **(B)** A discontinuity to the deep myo-aponeurotic connective tissue component of the T-junction (yellow arrow), Extension of the discontinuity into the myofascial connective tissue of the Biceps Femoris short head (blue arrow). **(C)** A discontinuity to the deep myo-aponeurotic connective tissue component of the T-junction (yellow arrow), A discontinuity to the superficial myo-tendinous connective tissue component of the T-junction (red arrow), Extension of the discontinuity into the myofascial connective tissue of the Biceps Femoris short head (blue arrow). **(D)** A discontinuity to the deep myo-aponeurotic connective tissue component of the T-junction (yellow arrow), A discontinuity to the superficial myo-tendinous connective tissue component of the T-junction (red arrow), Extension of the discontinuity into the myofascial connective tissue of the Biceps Femoris short head (blue arrow), Formation of an intermuscular haematoma between the long and short heads of the biceps femoris (pink arrow). **(E)** A discontinuity to the deep myo-aponeurotic connective tissue component of the T-junction (yellow arrow), A discontinuity to the superficial myo-tendinous connective tissue component of the T-junction (red arrow), Extension of the discontinuity into the myofascial connective tissue of the Biceps Femoris short head (blue arrow), Formation of an intermuscular haematoma between the long and short heads of the biceps femoris (pink arrow), Asynchronous movement (gapping/separation) of the long and short heads of the biceps femoris on dynamic ultrasound with a resisted isometric knee flexor contraction. BL, Biceps Femoris long head; BS, Biceps Femoris short head. (Created by the authors).

- A discontinuity (>1 mm) * involves the deeper myo-aponeurotic connective-tissue component only (Figure 1A).
- The superficial myo-tendinous connective-tissue component remains structurally intact.
- There is no extension into the myofascial connective tissue of either the long or short head, and no intermuscular haematoma is present.
- Dynamic ultrasound demonstrates preserved synchronous movement of the long and short heads during resisted isometric knee flexion.
*Diagnostic ultrasound achieves superior spatial resolution to MRI for tendon injury imaging, with axial resolutions of 0.1–0.5 mm and lateral resolutions of 0.2–1 mm using high-frequency linear transducers, compared to MRI's typical in-plane resolution of 0.4–1 mm, enabling finer depiction of fibrillar disruptions and partial tears (25).

### 8.1 Interpretation

This subtype represents a lower-grade structural lesion confined to the collagen-dominant aponeurotic layer. Mechanical continuity between the long and short heads is preserved, and prognosis is typically excellent with conservative management.

### 8.2 T-junction subtype 2

- A discontinuity involves the deeper myo-aponeurotic connective-tissue component with extension (>1 mm) * into the myofascial connective tissue of the biceps femoris short head laterally and/or long head medially (Figure 1B).
- The superficial myo-tendinous connective-tissue component remains intact.
- No intermuscular haematoma is evident, and synchronous movement of the long and short heads is maintained during resisted isometric knee flexion.
* High-frequency linear transducers (>10–20 MHz) enable diagnostic ultrasound to detect myofascial tears with sub-200 μm (0.2 mm) spatial resolution, manifesting as hypoechoic disruptions or irregularities within perimysial and fascial layers during dynamic scanning. This resolution surpasses conventional MRI capabilities for superficial fascia, facilitating distinction of subtle myofascial lesions from oedema-related alterations in muscular architecture (26).

### 8.3 Interpretation

Represents an aponeurotic lesion with limited myofascial extension but preserved mechanical coordination between the muscle heads. Prognosis and recovery are typically comparable to a conventional hamstring injury.

### 8.4 T-junction subtype 3

- A discontinuity involves both the deeper myo-aponeurotic connective-tissue component and the superficial myo-tendinous connective-tissue component (Figure 1C).
- The discontinuity extends into the myofascial connective tissue of the biceps femoris short head laterally and/or long head medially.
- No intermuscular haematoma is present, and synchronous movement of the long and short heads is preserved during resisted isometric knee flexion.

### 8.5 Interpretation

This subtype reflects a multi-layer lesion involving both structural components of the T-junction, but without disruption of dynamic coordination. Prognosis is good, though recovery is typically slower than Types 1–2 due to involvement of the myo-tendinous interface, which heals more gradually.

### 8.6 T-junction subtype 4

- A discontinuity involves both the deeper myo-aponeurotic connective-tissue component and the superficial myo-tendinous connective-tissue component (Figure 1D).
- The lesion extends into the myofascial connective tissue of the biceps femoris short head laterally and/or long head medially.
- An intermuscular haematoma * is present between the long and short heads of the biceps femoris.
- Dynamic ultrasound demonstrates preserved synchronous movement of both heads during resisted isometric knee flexion.

* Diagnostic ultrasound detects intermuscular haematomas at spatial resolutions of approximately 0.1–0.5 mm axially using high-frequency linear transducers (12–18 MHz), An intermuscular haematoma is defined as an acute anechoic or hypoechoic collections with peripheral displacement of muscle fibres (26).

### 8.7 Interpretation

Represents a high-grade, multi-layer lesion with associated intermuscular haematoma but preserved dynamic coordination. Although mechanical continuity remains, recovery is often prolonged due to the extent of tissue disruption and haematoma resolution.

### 8.8 T-junction subtype 5

- A discontinuity involves both the deeper myo-aponeurotic connective-tissue component and the superficial myo-tendinous connective-tissue component (Figure 1E).
- The discontinuity extends into the myofascial connective tissue of the biceps femoris short head laterally and/or long head medially.
- An intermuscular haematoma is present between the long and short heads of the biceps femoris.
- Dynamic ultrasound demonstrates asynchronous movement—evident as gapping or separation—between the long and short heads during resisted isometric knee flexion.

### 8.9 Interpretation

Represents a full-thickness, multi-layer disruption with loss of mechanical continuity across the T-junction. The presence of asynchronous motion suggests functional detachment between the muscle heads and may warrant consideration of surgical repair, particularly in cases of recurrent injury or failed conservative management. This finding has been proposed as a potential marker for surgical consideration, particularly where asynchronous motion persists despite conservative management or following re-injury. Asynchronous movement is characterised by divergent displacement of the biceps femoris heads, with the short head exhibiting subtle lateral translation and the long head demonstrating minimal medial translation. This discordant kinematics is accompanied by dynamic widening of the discontinuity at the interface between the long and short heads, consistent with progressive mechanical decoupling at the T-junction.

## 9 Discussion

### 9.1 Clinical interpretation and rehabilitation implications

The proposed five-type classification defines a clear continuum of structural and functional severity (Table 1). Subtypes 1–2 typically represent aponeurotic-dominant lesions with preserved coordination and predictable recovery. Subtype 3 reflects a transitional stage where myo-tendinous disruption begins to influence healing rate and load tolerance. Subtype 4 involves multi-layer injury with haematoma formation but maintained synchrony, requiring slower early loading and close ultrasound monitoring. Subtype 5 indicates functional dissociation between the long and short heads, often associated with mechanical instability and prolonged rehabilitation.

**Table 1:** Structural features defining T-junction subtypes as observed on ultrasound.

T-junction subtype	Myo – aponeurotic discontinuity	Myo-tendinous discontinuity	Myofascial discontinuity (long and or short heads)	Intermuscular haematoma	Asynchronous movement
Type 1	*✓*	**✗**	**✗**	**✗**	**✗**
Type 2	*✓*	**✗**	*✓*	**✗**	**✗**
Type 3	*✓*	*✓*	*✓*	**✗**	**✗**
Type 4	*✓*	*✓*	*✓*	*✓*	**✗**
Type 5	*✓*	*✓*	*✓*	*✓*	*✓*

In practical terms, rehabilitation progression should align with both the structural characteristics and dynamic findings of the injury. Ultrasound thereby functions not only as a diagnostic modality but also as a guide for load progression, prognosis, and return-to-play planning across the spectrum of T-junction injury severity.

### 9.2 Ultrasound-guided rehabilitation approach

The classification informs prognosis and management, but the overarching goal remains consistent: to restore coordinated function between the long and short heads of the biceps femoris while progressively reloading the injured connective tissue in a way that respects both biological healing and the demands of the sport. Recent experimental work confirms that this coordination is not purely neural but mechanical, with the short head actively modulating long-head strain through transverse load transfer across the T-junction (9, 10). Restoring that mechanical relationship is therefore a key rehabilitation target. Progression should be guided by ultrasound appearance, load tolerance, and functional recovery rather than fixed timelines (Table 2).

**Table 2:** Summary of rehabilitation characteristics, timelines, and other considerations following selected classifications of T-junction injuries.

Category	Type 2	Type 4	Type 5
Timeline	3–6 weeks	10–12 weeks	>12 weeks (often extended post-surgery or in recurrent cases)
Loading, strength & range progression	Early proximal loading progressing rapidly to distal. Isometric and eccentric work introduced from the outset, advancing as tolerance allows.	Proximal-dominant early phase; gradual distal reloading after haematoma resolution (∼3–4 weeks). Outer-range tolerance and reactive work emphasised from week 5–6.	Protective early phase. Short-range, low-rate contractions until continuity improves or surgical clearance is achieved. Gradual progression to lengthened, higher rate loading as synchrony returns.
Rotational control	Early reintroduction of anti-rotation and deceleration drills once pain-free.	Isometric anti-rotation early; controlled concentric rotation from ∼2–3 weeks; eccentric work delayed.	Isometric anti-rotation once comfortable; controlled concentric rotation from ∼3–4 weeks; eccentric work reserved for the latter stages.
Adjuncts (PRP, surgery)	Rarely indicated; emphasis on load progression and coordination.	Aspiration or PRP may be considered; surgery if recurrent or structurally unstable.	Surgery may be indicated for unstable injuries with detachment or persistent asynchrony, particularly in elite settings.

Early loading remains fundamental across all injury types (27). Timely mechanical input supports collagen alignment, preserves fibre architecture, and stimulates remodeling (28). Even in more severe subtypes, low-intensity or limited-range loading provides the essential biological signal for repair and mitigates atrophy or maladaptive adaptation (16).

Rehabilitation should aim to restore tolerance to late-swing loading and ipsilateral trunk rotation—two positions that impose high mechanical stress on the distal biceps femoris (29, 30). In the early phase, hip-dominant loading with external rotation can engage the long head while relatively offloading the distal interface. As healing progresses, knee-dominant and lengthened-range loading are reintroduced to restore tensile tolerance across the junction. This logic is essentially the inverse of that applied in proximal intramuscular tendon injuries, where hip-dominant loading is deliberately delayed protecting early healing (5).

That said, joint dominance is only one of several modifiable variables influencing tissue stress. Range, load magnitude, plane of movement, rate, and anticipation also influence strain distribution. Moreover, limited short-head activation remains essential to support junction remodeling and balanced collagen alignment, particularly in the deeper myo-aponeurotic layer (28, 31). Nakao et al. (10) have demonstrated this by showing that the short head regulates long-head strain through mechanical coupling; its controlled recruitment likely aids structural recovery. The goal, therefore, is not isolation but progressive re-integration of both heads under carefully managed loading conditions.

Across all subtypes, these controllable variables underpin safe and effective progression—from controlled, proximal-dominant isometrics to lengthened, high-rate, and reactive phases. In intermediate lesions (Type 3), early hip-dominant loading helps limit shear across the junction (32–34), with serial ultrasound used to confirm discontinuity filling and haematoma resolution before advancing knee-dominant and outer-range work. Week-to-week improvement in ultrasound appearance can act as a practical guide for progression. Key markers include reduction in haematoma size, restoration of fibrillar continuity (recorded as yes/no and the approximate size of any remaining discontinuity), and return of synchronous motion between the long and short heads on light prone contraction. These provide objective indicators that tissue tolerance is improving.

Using this as a periodic gauge of response allows loading to be adjusted appropriately. If these markers show steady improvement, progression in load intensity, contraction duration, contraction type, or running exposure can be justified. If they plateau, it likely indicates that current loading rate or magnitude exceeds tissue capacity, signalling the need to reduce volume or intensity. Persistent gapping on light prone contraction is a particularly important finding, suggesting that heavier eccentric work is not yet appropriate. As such, these ultrasound features can function as useful performance indicators to calibrate progression and ensure that load is aligned with the underlying tissue response.

Concurrent attention should also be given to the broader kinetic chain. Reduced control of the trunk (35), pelvis (36), hip, or ankle (37) can elevate hamstring load during running. Addressing these deficits early redistributes mechanical stress and improves tolerance to high-speed and deceleration tasks. Longer rehabilitation windows—as in Types 4 and 5—allow greater focus on these contributors while protecting the healing tissue.

Ultrasound is a valuable imaging modality for sports injury imaging, but several limitations must be acknowledged. Its diagnostic performance is inherently operator dependent, with image acquisition and interpretation closely tied to the examiner's training and experience, which can introduce variability and reduce reproducibility in less experienced hands. Image quality is further degraded in individuals with high adiposity, because increased soft-tissue depth attenuates the ultrasound beam and reduces the conspicuity of subtle lesions. Furthermore, accurate assessment of fine tendon and myofascial architecture generally necessitates high-frequency, non-handheld cart-based transducers. Additionally, ultrasound has a restricted ability to visualise deeper musculotendinous and intra-articular structures compared with MRI, making it less sensitive for small or deep-seated injuries.

Prospective validation of the proposed ultrasound-based classification for biceps femoris T-junction injuries should follow a multi-phase pathway, beginning with standardised rater training followed by interrater reliability assessment to establish diagnostic reproducibility. Subsequent longitudinal cohort studies in elite athletes would evaluate prognostic performance by correlating classification grades with validated outcomes such as return-to-play timelines and reinjury rates, benchmarked against existing MRI-based classifications schemes. Future work could integrate digital imaging tools, such as automated T-junction tracking software or AI-assisted ultrasound analysis for real-time dissociative motion detection, to enhance objectivity and scalability in clinical practice.

## 10 Conclusion

This ultrasound-based classification provides a reproducible foundation for diagnosing and managing T-junction injuries of the biceps femoris. By integrating static and dynamic ultrasound assessments, it captures structural disruption and functional behaviour in a clinically meaningful way that directly informs load progression and rehabilitation planning. Serial ultrasound during rehabilitation is advised to monitor healing and guide progression. Prospective validation across diverse clinical and surgical cohorts will be essential to determine whether this framework can standardise reporting and improve outcome prediction in this complex and under-recognised region.

## Data Availability

The original contributions presented in the study are included in the article/Supplementary Material, further inquiries can be directed to the corresponding author.
